# The Tumor Environment in Peritoneal Carcinomatosis and Malignant Pleural Effusions: Implications for Therapy

**DOI:** 10.3390/cancers17193217

**Published:** 2025-10-02

**Authors:** Paige O. Mirsky, Patrick L. Wagner, Maja Mandic-Popov, Vera S. Donnenberg, Albert D. Donnenberg

**Affiliations:** 1 Allegheny Health Network Research Institute, West Penn Hospital, 4800 Friendship Avenue, 2102 West Tower, Pittsburgh, PA 15224, USA; paige.mirsky@ahn.org (P.O.M.); patrick.wagner@ahn.org (P.L.W.); maja.popov@ahn.org (M.M.-P.); 2 Department of Surgery, Drexel University College of Medicine, 2900 W Queen Ln, Philadelphia, PA 19129, USA; 3 Department of Medicine, Drexel University College of Medicine, 2900 W Queen Ln, Philadelphia, PA 19129, USA; 4 Department of Cardiothoracic Surgery, Hillman Cancer Center, University of Pittsburgh School of Medicine, 5117 Centre Avenue, Pittsburgh, PA 15213, USA; donnenbergvs@upmc.edu

**Keywords:** peritoneal carcinomatosis, malignant pleural effusion, cellular therapy, immunotherapy

## Abstract

**Simple Summary:**

Peritoneal carcinomatosis (PC) and malignant pleural effusions (MPE) are two common manifestations of metastatic cancer. The native cavitary immune environment and the metastatic tumor interact in ways that promote aggressive tumor behavior, both recruiting and suppressing immune cells. The cellular and secretomic complexity of the cavitary tumor environment renders most currently available therapeutics ineffective. The unique cavitary tumor environment invites approaches that leverage the robust T-cell infiltrate while addressing the causes of local suppression of anti-tumor immunity.

**Abstract:**

Peritoneal carcinomatosis (PC) and malignant pleural effusions (MPE) are two common complications of cancers metastatic to the respective body cavities. A PC diagnosis indicates metastasis to the tissue lining the abdominal cavity and is most common in patients with gastrointestinal and gynecological cancers. It is often accompanied by ascites, an accumulation of serous fluid in the abdomen. MPE presents as the accumulation of fluid in the space between the lungs and chest wall. It is a common terminal event in patients diagnosed with breast cancer, lung cancer, lymphoma, and mesothelial cancers, and less commonly, in a wide variety of other epithelial cancers. Due to the aggressive nature of cavitary tumors, the outcome of current treatments for both PC and MPE remains bleak. Although PC and MPE are characteristically affected by different sets of primary tumors (lung/breast/mesothelioma for MPE and gynecologic/gastrointestinal for PC), their environments share common cytokines and cellular components. Owing to the unique cytokine and chemokine content, this environment promotes aggressive tumor behavior and paradoxically both recruits and suppresses central memory and effector memory T cells. The cellular and secretomic complexity of the cavitary tumor environment renders most currently available therapeutics ineffective but also invites approaches that leverage the robust T-cell infiltrate while addressing the causes of local suppression of anti-tumor immunity. Interactions between the heterogeneous components of the tumor environment are an area of active research. We highlight the roles of the immune cell infiltrate, stromal cells, and tumor cells, and the soluble products that they secrete into their environment. A more comprehensive understanding of the cavitary tumor environment can be expected to lead to better immunotherapeutic approaches to these devastating conditions.

## 1. Introduction

### 1.1. Peritoneal Carcinomatosis

Metastasis is responsible for approximately 90% of cancer deaths, making it the foremost cause of cancer mortality and morbidity [1]. Metastasis to the peritoneal [2,3] or pleural [4] cavities carries a particularly poor prognosis, owing to the unique intracavitary environment [5,6]. This article focuses on peritoneal carcinomatosis (PC) and malignant pleural effusions (MPE), forms of metastases in which cancer spreads to the peritoneum or the pleural cavity.

The peritoneal cavity surrounds the abdominal organs and is lined by a monolayer of mesothelial cells [7,8]. In healthy populations, the peritoneum contains 50–75 mL of serous fluid [9] and functions as a regulator for inflammatory responses [10]. PC develops as cancer cells migrate to the peritoneal cavity and adhere to the epithelial tissue of the peritoneum [7]. Here, cancer cells proliferate and trigger the process of angiogenesis, where tumor-associated blood vessels expand and support metastasis in the cavity [7]. Metastasis to the peritoneum is most commonly seen in cases of gynecological and gastrointestinal cancers, which can include colorectal, ovarian, pancreatic, and appendiceal cancers of origin [7,10,11]. PC denotes an advanced cancer status with an average survival of less than six months after diagnosis [7]. Patients typically experience aggressive tumor growth with additional symptoms of abdominal swelling, abdominal pain, dyspnea, and nausea as a result of a buildup of peritoneal fluid [12,13]. Eventually, most patients progress to develop bowel obstruction, malnutrition, cachexia, and death. The presence of malignant cells in peritoneal fluid is a key indicator of peritoneal carcinomatosis.

**Figure 1:**
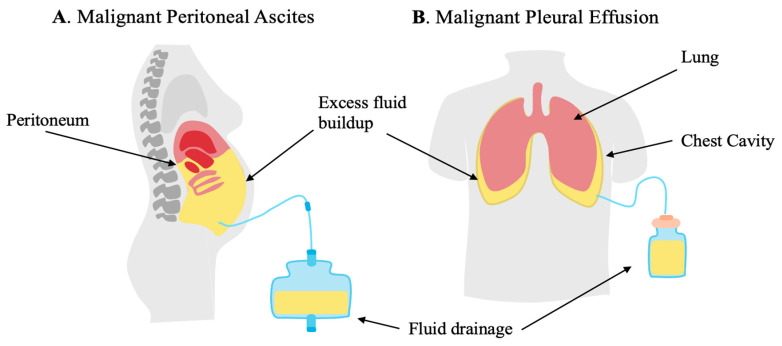
Malignant ascites (MAs) secondary to peritoneal carcinomatosis (PC) and malignant pleural effusions (MPE) are characterized by the buildup of excess fluid as a result of interactions of tumor, resident immune cells, resident stromal cells, and infiltrating immune cells. (**A**) MA forms in the peritoneal cavity. (**B**) MPE forms between the lung and chest wall, compressing the lungs.

The normal, uninflamed peritoneal cavity contains a cytokine and immune cellular profile distinct from that of the peripheral circulation [6]. Approximately 70% of patients with PC develop malignant ascites (MA) due to an influx of serous fluid resulting from tumor-induced increased vascular permeability or, less frequently, lymphatic obstruction [10]. A key indicator of vascular permeability includes high protein content seen in the MA fluid [13]. Increased levels of vascular endothelial growth factor (VEGF) have been strongly associated with the ability of proteins to travel across the vascular endothelium. Elevated VEGF is not observed within non-malignant ascites [13,14]. Secretion of VEGF by metastatic tumors in response to hypoxia promotes the genesis of disorganized and leaky small blood vessels, promoting the formation of MA [13]. Metastatic tumor also interacts with mesothelial cells and the normal immune components of the peritoneum to establish an environment that is both immunosuppressive [12,13] and favorable for aggressive tumor behavior [6]. Paracentesis is the most common procedure for relieving the symptoms of ascites (Figure 1) [13]. The procedure utilizes a catheter inserted into the peritoneal cavity to drain the excess fluid [12,13]. Although this may provide relief of abdominal swelling, paracentesis is considered palliative, as most patients will experience re-accumulation of fluid [12,13].

As the current treatment options for PC are limited, treating physicians most often resort to a combination of cytoreductive surgery and hyperthermic intraperitoneal chemotherapy (HIPEC) as the leading current treatment options for PC [7,10,13]. Cytoreductive surgery involves resecting as much visible tumor as possible from the abdominal cavity. This procedure can involve the removal of the affected organs, with procedures including omentectomy, oophorectomy, splenectomy, and multiple bowel resections [10]. Immediately following cytoreductive surgery, chemotherapeutic agents heated to approximately 42 °C are injected into the peritoneal cavity during the HIPEC stage of treatment [10,13]. A heated environment increases the cytotoxic effects of the chemotherapy agents, as tumor cells are more sensitive to high temperatures. The use of cytoreductive surgery and HIPEC has been shown to extend expected survival from 3 to 24 months to 40–62 months for eligible patients, yet these treatments are ultimately palliative and pose challenges with a significant risk of complications.

### 1.2. Malignant Pleural Effusions

Like the peritoneal cavity, the pleural cavity is a common site for metastases and carries a poor prognosis [15]. Located between the lung and chest wall, the pleural cavity is altered by a buildup of tumor cells and excess fluid, referred to as malignant pleural effusion (MPE). In the normal pleural space, the cavity is lubricated by a small volume of pleural fluid that is regulated by lymphatic drainage. Increased vascular permeability of the pleural cavity is often responsible for the buildup of excess pleural fluid. In this case, the fluid exhibits high protein levels and is referred to as an exudative effusion [16]. In contrast, transudative effusions are more commonly caused by an imbalance of hydrostatic pressure, which contributes to excess fluid accumulation [16]. MPE most commonly occurs in lung and breast cancer, accounting for 50–65% of all MPE cases, but can occur with a wide variety of cancers [4,15]. Additionally, MPE is found in up to 90% of patients diagnosed with malignant pleural mesothelioma (MPM), a cancer that forms in the membrane of the chest and lungs [17]. MPE diagnosis is often associated with dyspnea, pain, cough, and overall poor quality of life, with most diagnosed individuals surviving an average of 3–12 months [4,18].

Due to the decreased life expectancy, care is often palliative. Tumor excision is rarely indicated in malignant pleural effusions. The preferred and primary treatment for pleural fluid buildup is thoracentesis, a procedure that drains fluid from the pleural cavity (Figure 1) [4]. Thoracentesis provides relief of symptoms associated with MPE; however, fluid reaccumulates rapidly, requiring frequent intervention. For those who require frequent drainage (several times per week) for symptomatic relief, placement of an indwelling pleural catheter (IPC) is indicated [4,18]. IPCs are silicone catheters positioned between the pleural entry point and the skin exit site. Since the COVID-19 pandemic, home drainage, under the supervision of a trained household member or visiting healthcare worker, is increasingly common [19]. Chemical pleurodesis is another approach to palliation that is used less frequently because of the risk of inflammation, infection, and significant respiratory distress [4]. Pleurodesis involves injecting sclerosing agents such as talc, bleomycin, or tetracycline either by thoracoscopy or through an indwelling catheter. The chemical agents cause inflammation and adhesions between the chest wall and lung, thus preventing fluid from accumulating. In addition to drainage, patients usually receive systemic cytotoxic agents, small-molecule inhibitors, or endocrine therapy, depending on the tissue of origin. Despite such efforts, median survival was determined to be 5 months from initial diagnosis in a large series of malignant pleural effusions, including multiple tissues of origin [20].

In this review, we highlight the cellular components, secretomes, and relevant immune response mechanisms characteristic of the malignant peritoneal and pleural environments. Taken together, this information may serve to inform future immunotherapeutic approaches to MA and MPE.

## 2. Role of Immune Infiltrating Cells in Cavitary Malignancies

### 2.1. Immune Cell Infiltration

The peritoneum comprises visceral and parietal serosal membranes lining the abdominal cavity. The visceral peritoneum lines the abdominal organs, and the parietal peritoneum lines the inside of the abdominal cavity. They comprise a monolayer of mesothelial cells, joined by tight junctions, and, together, form a protective barrier [8]. Their primary function is to minimize friction when abdominal organs shift. The mesothelial cells sit atop a basement membrane and a layer of connective tissue that includes cells, blood vessels, and lymphatic vessels [21]. The normal peritoneal cavity has its own complement of resident immune cells, distinct from the peripheral circulation [22]. The resident immune cells are associated with innate immune response and the repair and maintenance of the peritoneal surface. The cellular contents of normal peritoneal fluid include macrophages (45%), T cells (45%), natural killer (NK) cells (8%), and B cells (2%) [23]. Similarly, the visceral and parietal pleura are lined with mesothelial cells and are bathed in a small volume of serous fluid containing macrophages (75%), lymphocytes (23%), previously shed mesothelial cells (1–2%), and neutrophils (1–2%) [24]. Since the healthy peritoneal and pleural cavities are sterile, constitutive immune functions are keyed to tissue repair and not effector responses, with macrophages favoring M2 differentiation [25,26].

When malignancies metastasize to or originate in the peritoneal or pleural cavity, the resident immune cells work to their advantage. Rather than attack the tumor, M2 polarized macrophages [27] and T_H_2 polarized T cells [28], normally involved in tissue maintenance and wound healing, form an environment conducive to tumor growth and aggressive behavior. Particularly, peritoneal and pleural M2 macrophages promote tumor growth, tissue remodeling, and angiogenesis, while suppressing anti-tumor adaptive immune responses [27,29]. They also promote the epithelial-to-mesenchymal transition (EMT) [30], a state that converts sessile epithelial tumor cells into a motile [31], drug-resistant [32], fibroblastoid phenotype [33]. A preponderance of infiltrating neutrophils, which are normally scarce in peritoneal and pleural fluid, is associated with unfavorable outcomes in peritoneal and pleural malignancies [34], which themselves carry a very poor prognosis. Immature neutrophils are the most prevalent type of myeloid-derived suppressor cells, a heterogeneous group of myeloid cells capable of suppressing adaptive immune responses [35].

### 2.2. T-Cell Exhaustion Versus Quiescence

T-cell exhaustion is defined functionally by the loss of proliferative capacity and effector activity. Exhausted T cells are recognized by the coexpression of multiple immune checkpoint molecules (ICM), including PD-1, CTLA-4, TIM-3, and LAG-3 [36]. Mechanistically, T-cell exhaustion results from chronic antigen stimulation and is mediated by epigenetic modifications [37]. Such DNA modifications, including methylation and histone modification, determine which genes can be transcribed and effectively close off the possibility of effector responses. In normal immunophysiology, immune exhaustion provides a means of limiting proliferation of individual T-cell clones, dampening effector responses, and limiting collateral damage to normal tissues when effector responses are no longer appropriate. However, chronic antigen stimulation occurring in infections that fail to resolve [38], as well as in the tumor microenvironment, where tumor-infiltrating cells are prevented from mounting an anti-tumor response [39], also leads to exhaustion. In these cases, immune exhaustion is counterproductive. The failure of exhausted T cells to return to quiescence after chronic antigen exposure is characterized by persistence of “activation molecules” including ICM, despite abrogation of effector responses [40]. Thus, it is critical to determine whether the immune cell infiltrate characteristic of malignant ascites and effusions is predominantly exhausted or if activated and quiescent T cells are also present.

Like exhausted cells, quiescent T cells are in a resting metabolic state, devoid of effector functions and out of the cell cycle. The key differences are that quiescent T cells do not express activation markers or ICM and can be activated upon encountering antigen in a permissive context that is free of external immunosuppressive signals. Activated effector cells also resemble exhausted T cells in that they express activation markers and ICM, but are proliferative with robust cytokine and cytolytic responses and high metabolic activity [41].

Naïve T cells are inherently quiescent, meaning that they are not actively replicating and await activation through receptor engagement. Notably, this state of low metabolic activity is distinct from regulatory T-cell-mediated tolerance or anergy [42]. Naïve T cells exit their quiescent state and differentiate into effector T cells upon antigen stimulation. Quiescence is partially maintained by cell-intrinsic signals, including tonic T cell receptor (TCR) signaling and interaction with forkhead box protein 1 (FOXP1) [42]. Tonic TCR signaling refers to the ability of T cells to maintain a continuous and weak signal in the absence of an antigen, maintaining the resting state [43]. Tonic TCR signals work in conjunction with interleukin (IL)-7, a cytokine crucial for T cell survival, to induce the expression of proteins that promote cell survival and maintain cellular viability [43]. The binding of IL-7 to its receptor IL-7R leads to an upregulation of molecules that prevent programmed cell death, including Bcl-2.

Quiescence is not limited to naïve T-cells and represents a major mechanism for maintenance of the central memory cell pool [44]. The majority of memory T cells exist in a G1 cell cycle state, where they are resting but positioned for rapid functional response and progression to cell cycle upon antigen encounter [45]. In breast cancer-derived therapeutic TIL cells, the influence of FOXP1 has been identified as a down-modulator of immune response and activity [46].

### 2.3. Evidence That Cavitary Infiltrating T Cells in Malignancy Are Quiescent Rather than Exhausted

T-cell quiescence is highly influenced by the environment in which the cells reside. Certain cytokines present in the TME, including transforming growth factor beta (TGFβ), may act as enforcers of quiescence by upregulating TGFβ receptor type 1 (TGFβR1) [47]. TGFβ is expressed at elevated levels in both the MPE [48] and MA [49] fluid, which may act as a silencer of T cells in these specialized tumor environments.

T-cell exhaustion is a state in which T-cell function becomes impaired as a result of prolonged stimulation. Exhausted T cells do not secrete effector cytokines and are positive for ICM. MPE T cells express low levels of the ICMs PD-1, PDL-1, LAG-3, TIM-3, CTLA-4, and TIGIT [5]. MPE T-cell unresponsiveness can be reversed in vivo through the administration of intracavitary IL-2 [50] and in vitro by stimulation with anti-CD3/anti-CD28 [5]. After activation and expansion in vitro, MPE T cells are cytotoxic to autologous tumor, indicating release from their quiescent state [5]. Similarly, intracavitary IL-2 administration resulted in increased T-cell proliferation and expression of granzyme B and interferon (IFN)*γ* expression by CD8+ T cells [50].

### 2.4. Mechanisms of Cavitary Immune Suppression

Normal peritoneal and pleural fluids have complex secretomes resulting from the interaction of resident immune cells and stromal cells. In normal peritoneal fluid, concentrations of soluble IL-6 receptor alpha (IL-6Rα), TGFβ, CCL2, epidermal growth factor (EGF), CXCL1, and CCL22 exceed 100 pg/mL [49]. These cytokines are believed to play a role in the maintenance and repair of the mesothelial lining. In cavitary malignancies, these cytokines are joined by CXCL2, IL-6, IL-8, IL-1RA, VEGF, and IL-10 [48]. Some cytokines are secreted directly by the tumor, but others by stromal and immune cells under the influence of the tumor [5]. In combination with the constitutive cavitary cytokines, they combine to form a maladaptive environment that is chemoattractive, immunosuppressive, and supportive of EMT.

### 2.5. Summary

The pleural and peritoneal cavities are lined by mesothelial cells that constitutively secrete cytokines, chemokines, and serous fluid, assisting in tissue repair and reducing friction between organs. In the normal pleura and peritoneum, the environments contain T cells, NK cells, B cells, and macrophages that are primarily skewed towards M2 polarization, which tend to favor tissue repair. In the presence of malignancy, M2 cells become suppressors of adaptive immunity and promoters of tumor growth and the EMT pathway, causing tumor cells to become increasingly invasive and resistant to therapy. Further, the malignant intracavitary environment suppresses immunity as T cells become dysfunctional and assume either an exhausted or quiescent state. Exhausted T cells can be identified by their chronic antigen stimulation and increased immune checkpoint expression, while quiescence is signaled by a reversible low-activity state. Evidence suggests that tumor-infiltrating T cells within the intracavitary environments are quiescent rather than exhausted, and can be reactivated by stimulatory cytokines such as IL-2. T cell quiescence is highly enforced by immunosuppressive cytokines, including IL-10 and TGFβ, that are present in the malignant intracavitary environment. These mechanisms of immunosuppression further lead to a more chemoattractive and tumor-supportive framework.

## 3. Key Cytokines of the PC and MPE Tumor Environment

### 3.1. The IL-6 Axis

Both IL-6 and its soluble receptor IL-6Rα are prominent in cavitary malignancies. IL-6 is a pleiotropic cytokine, a marker of cancer-mediated inflammation [51], and is secreted by tumor cells, pleural mesothelial cells, and stromal cells [52]. High serum IL-6 levels have been associated with poor patient outcomes in cancer patients of varying tumor types [53,54]. The classical signaling pathway requires the cis binding of IL-6 to the α-chain of its receptor to form IL-6/IL-6Rα, which further binds to the transmembrane protein IL-6Rβ, also known as gp130 [51]. Classical IL-6 signaling is limited to cells that express IL-6Rα, either induced (activated T cells, B cells) or constitutive (hepatocytes). Because gp130, the signal transducing component of the IL-6 receptor, is constitutively expressed on a wide variety of cell types, IL-6 trans-signaling can be facilitated by the soluble form of IL-6Rα (sIL-6Rα). Soluble IL-6Rα binds to IL-6 in solution, forming the IL-6/sIL-6Rα complex. This complex can bind to gp130-expressing cells, triggering downstream pro-inflammatory cytokine responses [55] and promoting aggressive tumor growth.

**Figure 2:**
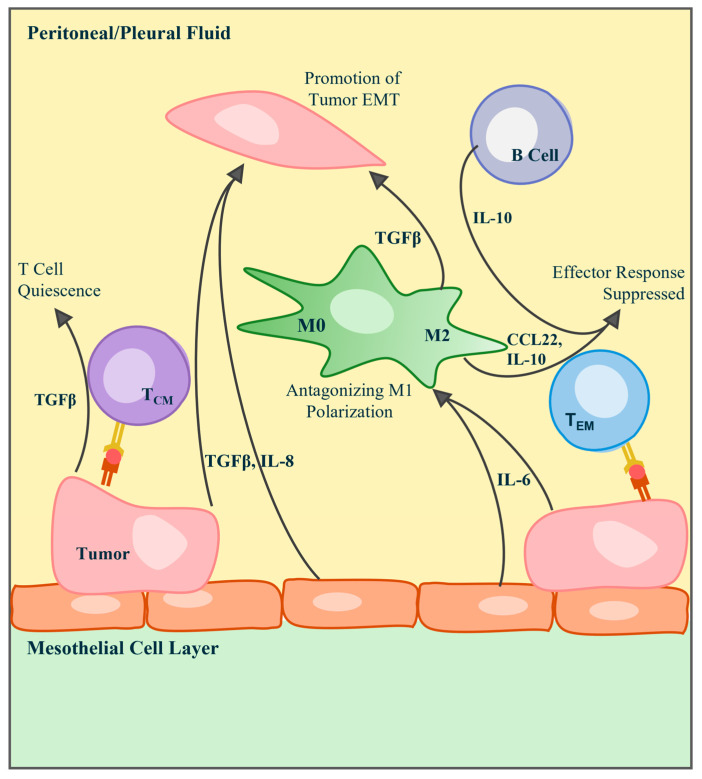
Immunological schematic of key cytokines, chemokines, and interleukins found in the pleural and peritoneal tumor environments. The epithelial-to-mesenchymal transition of tumor cells in the MPE or MA is promoted by the presence of TGFβ, secreted by tumor, mesothelial, and macrophage cells, and IL-8 secreted by tumor and mesothelial cells. T cells can kill tumor cells through direct MHC interaction, but their activity can be silenced or suppressed by molecules including TGFβ, IL-10, CCL22, and IL-10. Tumor-derived IL-6 aids in the stimulation of these immunosuppressive cytokines.

High levels of IL-6/IL-6Rα aid in the stimulation of immunosuppressive cytokine production, including IL-10 and IL-1RA, which are prominent in the MPE (Figure 2) [48,56]. The presence of IL-6/sIL-6Rα in MPE across cancers of different origins indicates the cytokine’s contribution to more aggressive tumor diagnoses and inflammatory responses [48]. Elevated levels of IL-6 have also been seen in patients diagnosed with PC. One study of patients with ovarian cancer metastatic to the pleural cavity observed significantly higher levels of IL-6 in MA samples than those with benign ovarian tumors [57]. The study also noted that the presence of IL-6 may act as a predictive biomarker, as patients expressing higher levels of the cytokine had shorter survival times [57]. Along with IL-6, sIL6-Rα has also been seen at elevated concentrations in the peritoneal fluid of patients with PC [49].

### 3.2. IL-10

IL-10 serves a number of complex functions in the cavitary environment and is frequently elevated in the serum of patients with a variety of cancer diagnoses [58]. IL-10 is a pleiotropic cytokine reported to play opposing roles in cancer [59]. Several cell types have been identified as IL-10 producers, including T_H_ cells, B cells, Treg, NK cells, and APCs [58,60]. IL-10 activity was originally described in relation to the downregulation of the T_H_1 cytokines IL-2, tumor necrosis factor (TNF)-α, and IFN*γ* and as a factor that is crucial to anti-inflammatory responses [58]. Thus, IL-10 is a self-regulatory component protecting against autoimmunity [61]. Despite its pleotropic nature, in the cavitary tumor environment, the major role of IL-10 appears to be to blunt immune effector responses (Figure 2) [58,59].

### 3.3. TGFβ

The transforming growth factor-β (TGFβ) group serves many immune response functions and comprises three isoforms, TGFβ1, TGFβ2, and TGFβ3 [62]. The pleiotropic nature of TGFβ is understood to be a result of the numerous contexts in which the cytokine can be found. TGFβ is central to T cells and B cells of the adaptive immune system and regulates maturation and activation [62]. The cytokine has also been implicated in tissue repair and wound healing [63] and likely plays a similar role in the maintenance of the normal cavitary environment. In the cavitary tumors, TGFβ is produced by cancer cells, macrophages, and stromal cells. Elevated levels of TGFβ inhibit T cell differentiation to effector cells [62], interfering with tumor-specific responses. TGFβ also promotes EMT [64], a pathological consequence of its physiologic role in wound healing (Figure 2).

A study including 30 patients with MPM demonstrated significantly higher concentrations of TGFβ in MPE samples compared to non-malignant effusion samples [65]. Increased levels of TGFβ were correlated with more advanced disease staging as well as poor survival. TGFβ, specifically the isoform TGFβ1, is among the cytokines that are consistently elevated across MPE of multiple cancer types [48].

### 3.4. IL-1RA

The IL-1 receptor antagonist IL-1RA is a potent inhibitor of members of the IL-1 family, key cytokines in pro-inflammatory reactions. IL-1 plays a major role in T-cell effector responses, licensing committed effector cells to produce IFNγ, IL-17, and other cytokines [66]. In cavitary malignancies, IL-1RA may enforce quiescence of infiltrating central memory cells.

### 3.5. Cellular Mechanisms of Cavitary Immunosuppression

Regulatory T cells (Treg) have been demonstrated to mediate suppression of anti-tumor responses in a variety of malignancies [67]. Using both the GeoMx platform and conventional flow cytometry, we have failed to detect Treg among pleural infiltrating T cells in breast cancer [5]. However, Budna et al. reported an increase in the number of Treg in malignant effusions compared to benign effusions [68]. Elevated levels of Treg have also been detected in MPA, compared to peripheral blood [69]. Repeated efforts on our part, using a well-validated flow cytometry panel [70], have failed to confirm a prominent role for Treg in MPE. Regulatory B cells (Breg) exert their suppressive effect through IL-10 secretion [71] and are a potential candidate for suppression of anti-tumor immunity in cavitary malignancies [72].

Neutrophils are usually not abundant in MA or MPE. Their presence may signal empyema, a bacterial infection associated with repeated drainage. In the absence of infection, a high neutrophil to lymphocyte ratio (>0.7) is predictive of poor survival [73]. Myeloid-derived suppressor cells (MDSC) have been characterized as immature myeloid cells and myeloid progenitor cells [74]. MDSCs can suppress T-cells through depletion of L-arginine and L-cystine, by induction of oxidative stress through reactive oxygen species [75], and through secretion of pro-inflammatory cytokines such as IL-6 and IL-8 [76]. MDSCs have been identified in PC, where they are reported to contribute to immunosuppression and tumor progression [77].

### 3.6. Summary

Cavitary malignancies contain a network of cytokines and cells that promote tumor growth, EMT, and immunosuppression. Tumors, stromal cells, and mesothelial cells produce IL-6, a pleiotropic cytokine linked to an inflammatory environment and poor prognoses at elevated concentrations. Produced by APCs, B cells, and NK cells, IL-10 has been identified as a largely pleiotropic cytokine but primarily a downregulator of immune effector responses in the malignant setting. TGFβ is consistently elevated in malignant effusions and inhibits T cell activation and drives EMT, both of which correlate with advanced disease status. Several cellular mechanisms, including those imposed by Bregs and other IL-10-secreting cells, actively suppress anti-tumor immunity. When present, neutrophils and MDSCs further promote immunosuppression through cytokine secretion and oxidative stress. Taken together, the immune cells and cytokines of intracavitary malignancies establish a maladaptive and tumor-promoting environment.

## 4. Tumor–Stromal Interactions

The stroma comprises connective tissue, blood vessels, lymphatics, nerves, and extracellular matrix (ECM) that surround and support the function of a specialized organ [78]. Cancer–stroma interactions are characterized by the interplay of tumor cells and the surrounding non-cancerous tissues and cells. The well-vascularized stroma serves as the tumor’s source of nutrients and clears waste products [79]. The stroma of the pleura and peritoneum contains fibroblasts, endothelial cells, immune cells, and their associated ECM [8,80]. In both the peritoneum and the pleura, the stroma includes a monolayer of mesothelial cells in a cobblestone formation joined by tight junctions [81].

Cancer-associated fibroblasts (CAFs) refer loosely to a group of non-neoplastic cells of diverse origin that assume a fibroblastoid morphology under the influence of a tumor [82,83]. As such, they are both influenced by the tumor microenvironment, and themselves serve to condition the microenvironment through both paracrine and juxtacrine [84] mechanisms. Production of cytokines by CAFs influences tumor proliferation and angiogenesis, ECM remodeling, immune cell recruitment, and tumor metastasis [82]. Specifically in PC, CAFs are produced from mesothelial cells undergoing a mesothelial-to-mesenchymal transition (MMT), paralleling the EMT of epithelioid tumor cells. As tumor cells transition to a mesenchymal state, their ability to adhere to and invade the peritoneal stroma and metastasize to extraperitoneal sites is heightened [85]. The processes of MMT and EMT are primarily induced by cytokines TGFβ1 [64] and IL-6 [84]. In vitro and in vivo experiments explored the blockade of TGFβ1, and both demonstrated that interference with the TGFβ1 pathway prevented mesothelial cell mechanisms that lead to peritoneal metastases [86]. Another study observed the ability of macrophages originating from hematopoietic stem cells to differentiate into CAFs in the context of both MPE and MA [87]. Using gene ontology, the group concluded that TGFβ produced by the MPE and MA environments promotes the differentiation of macrophages into CAFs. Further, the differentiated cells are more inclined to promote tumor cell proliferation than their macrophage precursors.

CAFs have been identified as inducers of increased ECM synthesis, as well as ECM remodeling by proteinases, ultimately promoting tumor progression [78,88]. The ECM has many components, including proteoglycans, hyaluronic acid, fibrous proteins, growth factors and cytokines, antibodies, and numerous resident cell types [78]. Remodeling of the ECM further occurs through the production of the fibrous protein collagen, which is heavily influenced by CAFs. ECM stiffness, defined by the degree of collagen deposition, is associated with progression in PC resulting from colorectal cancer [88].

## 5. Immunotherapy of PC and MPE

Current treatment options for PC include surgery, chemotherapy, and targeted therapy, often in combination, and have extended median survival from 3 to 24 months to 40 to 62 months [10]. Intraperitoneal chemotherapy is often used in combination with surgical cytoreduction [10]. Although patients with MPE usually receive systemic treatments based on the tissue of origin of their primary cancer, survival is poor and correlates with performance status at the time of effusion onset [20]. Several palliative options currently exist to relieve symptoms of MPE, including thoracentesis, placement of an indwelling tunneling catheter, or pleurodesis with sclerosing agents such as talc or bleomycin [15]. Similarly, paracentesis is often used to reduce fluid volume in MA, in some cases requiring placement of an indwelling drain.

Strategies for regional immunotherapeutic approaches in PC and MPE are gaining momentum with the increasingly mainstream utilization of immune checkpoint blockade, adoptive cellular therapy (ACT), chimeric antigen receptor T cell (CAR-T) therapy, bispecific antibodies, cytokines, and oncolytic viruses in other clinical scenarios. In some studies, therapeutic agents have been administered directly into the peritoneum or pleura. With the exception of CAR-T and other receptor-engineered ACT, immunotherapy is predicated on the notion that cancer patients naturally mount an adaptive immune response to tumor neoantigens, but this response is actively suppressed by the tumor. Thus, cancer immunotherapies aim to overcome such suppressive effects, unleashing dormant anti-tumor responses. Ongoing immunotherapeutic trials are highlighted in Table 1.

### 5.1. Antibody-Based Immunotherapy of PC and MPE

Intraperitoneal immunotherapy with agents such as IFNα [89] and picibanil [90] has been investigated since the 1980s. In some cases, these immunotherapeutics have been used in conjunction with intravenous chemotherapeutics. Regional intracavitary administration via intrapleural or intraperitoneal injection is an appealing treatment option for MA and MPE. As with conventional chemotherapy, regional delivery approaches have the advantage of maximizing dose tolerance at the site of intended activity, while reducing the risk of systemic toxicity, especially in the case of high molecular weight drugs such as antibodies and cytokines [52]. The cellular heterogeneity and complex combination of cytokines in both the peritoneum and pleura provide ample targets for rational therapeutics [91], as we and others have recently hypothesized [7,52].

#### 5.1.1. Anti-PD1

Nivolumab is an immune checkpoint inhibitor targeting the PD-1 molecule to prevent binding to PD-L1 and PD-L2, preventing cancer cells and cancer-associated macrophages from inducing apoptosis in immune effector cells [92]. The ATTRACTION-2 trial (N = 493) was a phase 3 randomized study of nivolumab in gastro-esophageal junction cancer patients who did not respond to two previous lines of chemotherapy [93]. Twelve- and 24-month survival were 87.1% and 61.3%, respectively, in patients who received nivolumab. This clinical trial, among others, led to the approval of nivolumab for patients with gastric cancer. Currently, there is limited data to support the intracavitary administration of single-agent anti-PD-1 antibodies.

Sintilimab, an anti-PD-1 monoclonal antibody (mAb) with reportedly higher binding affinity than nivolumab [94], was administered to patients with metastatic non-small cell lung cancer (NSCLC) Via intrapleural injection [95]. Of the nine patients who were enrolled in the study, seven patients experienced a significant effusion volume decrease after 5 weeks. After another 10 weeks of intrapleural sintilimab, the study reported a 66.7% control rate of MPE. Another study attempted to administer a single 40 mg intrapleural dose of nivolumab to patients with metastatic NSCLC. Although the group had previously demonstrated the safety of intrapleural nivolumab, they found that a single dose was ineffective in the treatment of MPE due to NSCLC [96,97]. Although current evidence suggests that local anti-PD-1 therapy is only marginally effective as a single agent, its use in combination with other modalities, such as ACT, remains promising.

#### 5.1.2. Anti-CTLA-4

CTLA-4 is an immune checkpoint molecule that inhibits the binding of CD28 to CD80/CD86, thereby interfering with the antigen-presenting cell-mediated second signal required for T-cell activation [98]. The anti-CTLA-4 monoclonal antibody ipilimumab was initially tested in the setting of metastatic melanoma [99]. In other cancer types, ipilimumab is regarded as ineffective when used as a monotherapy and is therefore frequently used in conjunction with a second agent [100,101].

Success rates of ipilimumab treatment have been largely improved by the addition of nivolumab [100]. The two drugs are often used together to treat a number of cancer types. In a phase 1b trial consisting of 18 patients with recurrent gynecological malignancies and PC, nivolumab plus ipilimumab were administered via intraperitoneal injection [91]. The study found that approximately 19% of patients had an objective response to the therapy, with one patient exhibiting a complete response.

#### 5.1.3. Anti-VEGF

Bevacizumab is a mAb targeting VEGF, a pro-angiogenic factor and inducer of vascular permeability. VEGF is recognized as an important factor in the establishment and support of cancer metastasis. Although increased levels of serum VEGF are often seen in patients with a variety of cancers, VEGF levels in MPE/MA fluid are far greater [102] and may contribute to both tumor vascularization and fluid imbalance [103].

Intrapleural bevacizumab was assessed in patients with NSCLC metastatic to the pleura. Twenty-seven patients received bevacizumab through an intrapleural catheter, while 122 had catheter placement without bevacizumab. In patients with actionable mutations (ALK/ROS1 fusions, EGFR), overall survival was marginally better in patients who received intrapleural bevacizumab (*p* = 0.045) [104].

A large study of 1235 patients observed the addition of bevacizumab to palliative chemotherapy regimens in patients diagnosed with colorectal cancer with PC [105]. Approximately 35% of the patients received bevacizumab plus chemotherapy, while the remainder received chemotherapy alone. The addition of bevacizumab to the care regimen resulted in a small but statistically significant improvement in survival, extending the patients’ lives an average of 3.5 months [105]. Clinical trials of intraperitoneal bevacizumab are ongoing. The REZOLVE study, a phase II trial, assessed intraperitoneal administration of bevacizumab in patients with epithelial ovarian cancer. After the drug administration, the time between paracentesis intervals increased by 4.29-fold [106]. Intraperitoneal administration of bevacizumab has been largely determined to be safe and effective in reducing fluid accumulation, but did not affect survival [106,107].

#### 5.1.4. Anti-IL6 Receptor

The anti-IL-6Rα drug tocilizumab was first approved for the treatment of moderate to severe rheumatoid arthritis [108]. Tocilizumab binds to both soluble and membrane-bound forms of IL-6Rα to inhibit IL-6-mediated functions. The presence of soluble IL-6Rα mediates IL-6 trans-signaling, which greatly increases the cell types capable of responding to IL-6 stimulation [51], amplifying inflammatory pathways that lead to EMT and aggressive tumor behavior [48,84]. As a result, targeting the IL-6/IL-6R axis of cancer has become the rationale for tocilizumab administration [5,11,48,52].

Soluble IL-6Rα is present at high concentration in normal pleural and peritoneal fluids [49], but IL-6 is normally absent. The intracavitary secretion of IL-6 by tumor and tumor-associated macrophages, in combination with soluble IL-6Rα trans-signaling, polarizes the intracavitary environment for aggressive tumor behavior and suppression of anti-tumor immunity. Intracavitary administration of the receptor antagonist tocilizumab has been of recent interest to condition the intracavitary tumor environment by locally suppressing responses to IL-6. RIOT2 is a phase I dose escalation trial in which patients with either MPE or MA receive intrapleural or intraperitoneal tocilizumab (Table 1) [109]. This ongoing trial is designed to test the safety of intracavitary tocilizumab also includes ancillary pharmacokinetic studies and tracks the effect of tocilizumab on pleural and peritoneal fluid volume.

### 5.2. Bispecific Antibodies

While mAbs target a single antigen, bispecific antibodies are engineered with the ability to bind to two different antigens [110]. Several bispecific antibodies have been approved for the treatment of cancer, with a select few being tested as treatments for MPE/PC.

A bispecific rat-mouse hybrid antibody with trifunctional properties, known as catumaxomab, has been assessed in both MPE and PC diagnoses. Being both bispecific and trifunctional indicates the antibody’s ability to target multiple cell types simultaneously [110,111]. The antigen-binding region of the bispecific antibody targets the epithelial marker EpCAM and the T-cell receptor CD3, while the Fc portions bind to the Fc gamma receptors I/III on macrophages, promoting T cell-mediated lysis, antibody-dependent cytotoxicity, and phagocytosis [111]. Catumaxomab has been assessed in several phase I/II clinical trials, including the treatment of PC in patients with ovarian, gastric, breast, pancreatic, colon, and endometrial cancers [111,112,113]. A study of 258 patients with varying epithelial cancers demonstrated the safety of intraperitoneal administration and showed that catumaxomab significantly increased the time between paracenteses. However, it did not significantly improve survival. In addition to its use in PC, catumaxomab was also assessed in patients with NSCLC and MPE [114]. Effusion samples of patients enrolled in a phase I/II clinical trial showed immunologic response and reduction in tumor cells present in MPE [114]. Despite evidence of safety and efficacy, this drug was voluntarily withdrawn from both the US market and the EU markets for commercial reasons [110].

More recently, phase I/II clinical trials have been evaluating a humanized anti-EpCAM/anti-CD3 bispecific antibody currently referred to as M701 [115,116]. The safety and efficacy of M701 are being assessed in cases of both MPE and MPA. A phase II study recruited 84 patients with MA from gastric, colorectal, and ovarian cancers to be randomized to one arm using paracentesis plus intraperitoneal M701 and the second arm using paracentesis alone [115]. Median time of drainage in patients receiving M701 increased from an average of 23 days to 75 days [115]. Additionally, 6-month overall survival increased from 12.6% to 32.2% in patients who received M701 [115]. Intrapleural administration of M701 for patients with NSCLC presenting with MPE is being assessed in a phase Ib trial [116]. The treatment regimen has an acceptable safety profile, but a further assessment of its efficacy is being studied in a phase II trial [116].

### 5.3. Oncolytic Viruses

Oncolytic viruses are a class of cancer therapeutics that selectively exploit properties of cancer cells, while sparing healthy cells, and are considered to be immunotherapeutic inasmuch as viral infection of cancer cells induces the expression of PAMPs, pathogen-associated molecular patterns that are perceived as “danger signals.” PAMPs stimulate an adaptive immune response to viral antigens [117,118]. Moreover, oncolytic viruses can be specially designed to recognize tumor-associated receptors, including EGFR, human epidermal growth factor receptor 2 (HER2), and the adenovirus fiber knob [119], or can be designed to preferentially infect rapidly proliferating tumor cells by knocking out viral proteins such as viral thymidine kinase or vaccinia growth factor [120]. Oncolytic viruses can be armed with cytokines, secreted by infected cells, to amplify the immune response to viral and tumor antigens. Novel membrane-bound cytokines, such as membrane-bound IL-12, localize cytokine activity to the surface of the infected tumor cell and avoid systemic toxicity [121]. Several oncolytic viruses have been tested in clinical trials. These include vaccinia virus [122], reovirus [123], adenovirus [124], and modified herpes simplex virus [125]. In a phase I trial, intraperitoneal administration of vaccinia virus GL-ONC1 for the treatment of PC, the drug was well tolerated, and no viral shedding was detected [126].

Many of the studies on oncolytic viruses for the treatment of MPE and PC are currently being tested in mouse models. One study employed a xenograft model of lung adenocarcinoma MPE treated with a tumor-specific vaccinia virus GLV-1h68, which led to a complete response in 50% of animals [127]. Oncolytic viruses can be effectively combined with other immunotherapeutic modalities such as immune checkpoint inhibitors, cytokines, monoclonal antibodies, or adoptive cellular therapeutics [118]. In the xenograft model of lung adenocarcinoma, the addition of a single-chain antibody against VEGF in the vaccinia construct further increased efficacy [127]. Similarly, IL-15-armed vaccinia virus increased survival in mouse models of colorectal and pancreatic cancer PC [128], and addition of an immune checkpoint inhibitor to vaccinia virus JX-594 resulted in the complete elimination of PC and MA in a proportion of mice injected intraperitoneally with MC38 colon cancer cells [129].

### 5.4. Summary

Current treatment options for PC and MPE traditionally involve surgical intervention, chemotherapeutics, and some targeted therapies, but survival outcomes for these diagnoses remain poor. Patients usually require palliative procedures, such as paracentesis, thoracentesis, and indwelling catheter placement, all of which are designed to manage fluid buildup, but typically do not improve long-term patient outcomes. Effective immunotherapies are actively being assessed, and in some cases, being directly administered to the intracavitary pleural or peritoneal environment to improve efficacy and reduce risk of toxicity. Immune checkpoint inhibitors such as nivolumab, sintilimab, and ipilimumab have shown promise in combinatorial settings. Bevacizumab has been shown to reduce fluid accumulation and marginally improve survival rates. Other drugs targeting key cytokines, including anti-IL-6R therapy (tocilizumab), are being assessed for safety and efficacy in early-stage clinical trials. Additionally, bispecific antibodies under investigation have provided early evidence of extended time between fluid drainage and have even improved survival rates in some settings. Various oncolytic viruses have also been tested in early clinical trials, with some showing promising safety and efficacy results, especially when combined with other immunological interventions.

## 6. Cellular Therapeutic Strategies for MPE and PC Treatment

Cellular therapeutics involve the use of ex vivo-expanded T cells or NK cells to treat diseases by exploiting native and engineered immune specificity. The field of cellular therapeutic-based treatments is rapidly growing and is expected to improve the treatment of many conditions, including metastatic cancer.

### 6.1. Tumor-Infiltrating Lymphocytes

Tumor-infiltrating lymphocytes (TIL) represent the immune cells found within the tumor environment [130]. As such, a proportion of TIL can be demonstrated to be specific for tumor neoantigens resulting from specific mutations, translocations, and fusions [131]. TIL are expanded from tumor tissue fragments in the presence of high-dose IL-2, under the premise that tumor-specific T cells will proliferate preferentially because their receptors are engaged by neoantigens. After this initial expansion, TIL are rapidly expanded in the presence of IL-2 and anti-CD3 antibody. Patients are given lymphodepleting chemotherapy (LDC) before the administration of the TIL product to elicit lymphoproliferative cytokines [132]. This, along with systemic IL-2 given at the time of TIL administration, is necessary for TIL survival. TILs are currently only Food and Drug Administration (FDA)-approved for the treatment of metastatic melanoma, but many other indications are currently the subject of clinical trials. In the 1990s, a pilot study was conducted to assess the safety of intraperitoneal TIL co-administered with intraperitoneal IL-2 in patients with advanced epithelial ovarian carcinoma [133]. LDC was not given. It appears the study was abandoned when no evidence of a measurable response was found. More recent studies have emphasized the critical role played by LDC [134] and concluded that tumors that appear to be immunologically cold can be rendered responsive to TIL by coadministration of immune checkpoint blockers [135,136,137].

### 6.2. CAR-T Cell Therapy

Unlike TIL, which relies on naturally occurring adaptive anti-tumor immunity, CAR-T cell therapy involves the use of synthetic chimeric antigen receptors (CAR) to enable T cells to recognize surface antigens present on tumors. CARs are transduced into peripheral T cells by vectors that encode four major components: an extracellular antigen-sensing domain, an extracellular hinge domain, a transmembrane domain, and an intracellular signaling domain [138]. The extracellular domain is made from the antigen-binding domain of a monoclonal antibody targeting a particular protein. The intracellular domain provides TCR and costimulatory signaling to initiate activation and effector status of the CAR-T cells [138]. Unlike TIL, which can recognize peptides from mutated intracellular proteins displayed on the cell surface by major histocompatibility complex (MHC) molecules, CAR-Ts are limited to cell surface proteins, few, if any, of which are truly cancer-specific. CAR-T tumor specificity depends on the tolerability of on-target off-tumor responses. For example, CD19 is expressed on most B-cell malignancies, but is also expressed on normal B cells. Anti-CD19 CAR-T therapy, which has resulted in salvage cures in B-cell acute lymphoblastic leukemia (B-ALL), B-cell non-Hodgkin lymphoma, and chronic lymphocytic leukemia (CLL) [139], is tolerable because the loss of normal B cells is compensated for by chronic administration of intravenous gamma globulin. Responses to other CAR-T targets, such as mesothelin [140] and EGFR [141], may be tolerated because these molecules are expressed at higher density on tumor cells compared to normal [142] or are conformationally distinct on tumor [143]. While CAR-T has proven efficacy in B-cell malignancies, the FDA has not yet approved this therapeutic for any other indication. Thus, most available data assessing the efficacy of CAR-T treatment in cancers with PC/MPE are primarily from animal models.

Treatment of PC with CAR-T cell therapy was initially investigated by the Katz group, which used a murine model to demonstrate PC as a complication of colorectal cancer [144]. CAR-Ts targeting the carcinoembryonic antigen (CEA) protein related to gastrointestinal cancers were administered via intraperitoneal or intravenous routes. CAR-Ts were only detected within the peritoneal cavity when administered by intraperitoneal injection and were associated with a reduction in peritoneal tumors. A second murine study used tumor-associated glycoprotein 72 (TAG72)-specific CAR with a 4–1BB intracellular signaling domain to target ovarian cancer PC [145]. Mice receiving intraperitoneal TAG72-specific CAR-T cells exhibited tumor growth reduction and longer survival times.

Similarly, Adusumilli et al. have explored intra-pleural delivery of mesothelin-specific CAR-T cells [140] for the treatment of MPM, a highly aggressive tumor type that frequently presents with MPE [146]. Mesothelin is a cell-surface protein that is highly overexpressed by mesothelioma, lung, breast, pancreatic, and ovarian cancer cells [147]. These investigators showed that the intra-pleural delivery route was more effective than intravenous administration; among 21 treated patients, 14 of whom also received anti-PD-1 therapy. Two achieved a complete metabolic response by PET CT after combination therapy [147].

Although these cellular therapeutics appear to have promise for treating patients with MPE/PC, it remains to be determine whether the theoretical advantages of regional delivery can outweigh the known potential for toxicity with CAR-T therapy, including cytokine release syndrome (CRS) [148,149] and on-target, off-tumor toxicity (i.e., immune destruction of normal cells that physiologically express the target molecules CEA and mesothelin [150,151].

### 6.3. Intracavitary TIL Cell Therapy

Intraperitoneal and intrapleural TIL can be isolated from MPE or MA fluid. In MPE, a single drainage can yield up to 10^9^ T cells [152]. A recent study from China assessed the use of intrapleural or intraperitoneal TIL therapy versus cisplatin chemotherapy for the treatment of MPE/MA [153]. A total of thirteen patients with MPE or MA were enrolled in the study and given either the TIL therapy or cisplatin after fluid drainage. TIL were derived from pleural-infiltrating T cells and were administered without LDC or IL-2. Despite the small number of subjects, progression-free survival was significantly longer in the TIL group (63 Vs. 143 days, *p* = 0.002). This result is remarkable both for the clear difference between treatment groups and the increased time of progression-free survival.

### 6.4. Fast TIL

**Figure 3:**
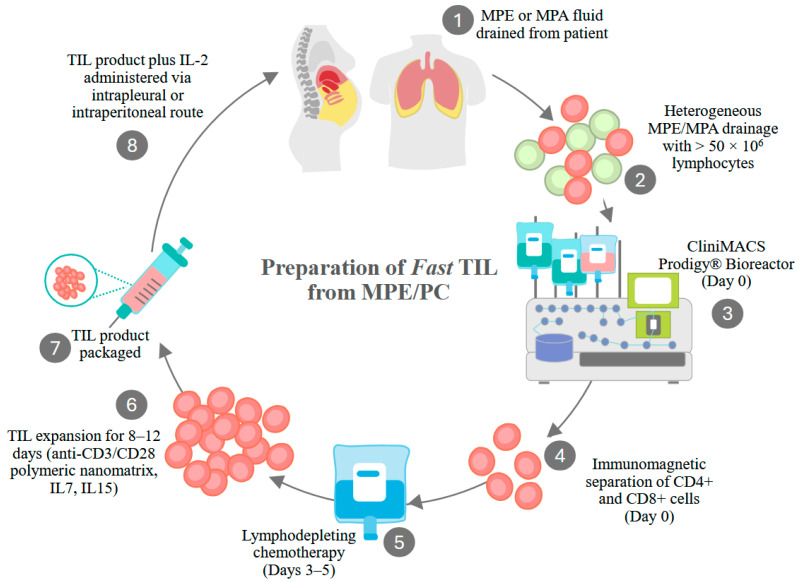
Generation of Fast TIL from MPE and PC fluid. Fast-TIL received an Investigational New Drug (IND 30892) designation for use in a Phase I clinical trial of multiple cancers metastatic to the pleura. Drained pleural fluid contains a variety of cell types (lymphocytes, macrophages, tumor cells, red blood cells, neutrophils, mesothelial cells). At least 50 × 106 lymphocytes must be recovered for processing to proceed. Cells are applied to the Miltenyi ClinMACS Prodigy, where CD4+ and CD8+ cells are immunomagnetically selected. Selected cells are then transferred to the cell culture/cell washing chamber, where they are activated by anti-CD3/CD8 nanomatrix particles. Cells are cultured in the presence of IL-7 and IL-15 for 8 to 12 days, washed, and resuspended in the final formulation medium. Patients receive lymphodepleting chemotherapy during the cell culture period. Prior to intrapleural infusion, the drug product undergoes release testing (absence of endotoxin, bacterial, fungal, or mycoplasma contaminants, cell purity, and viability). Therapeutic cells and IL-2 are coadministered through a pleural catheter. A clinical trial in PC/MA is also anticipated.

Investigators at the University of Pittsburgh School of Medicine and the Allegheny Health Network have jointly developed a pleural-infiltrating T-cell-derived ACT product [5,6] and have obtained an Investigational New Drug (IND) designation from the FDA to test this product in a variety of epithelial cancers metastatic to the pleura [152]. Starting with a minimum of 50 × 10^6^ pleural infiltrating T cells, Fast TIL refers to the short time required to expand cells to a therapeutic dose. After lymphodepleting chemotherapy, the product will be co-administered intrapleurally with IL-2 (Figure 3). Because cavitary malignancies exist in an environment that is highly immunosuppressive and conducive to aggressive tumor behavior, cavitary conditioning with anti-cytokine drugs or cytokines may be required to achieve the optimal performance of cellular therapeutics [52].

### 6.5. Summary

Cellular therapy is a rapidly evolving field with significant recent advancements for the treatment of various cancer types. TILs and CAR-T cell therapies are promising strategies for the treatment of PC and MPE. CAR-T cell therapies have been developed to engineer T cells to target specific tumor-associated proteins, such as mesothelin or CEA. Pre-clinical models and early-phase trials have shown promising results for the intracavitary administration of CAR-T, TILs are manufactured by expanding tumor-specific T cells from patient samples. While TIL therapy has been FDA-approved for use in melanoma patients, clinical trials are ongoing for other diagnoses, including intracavitary tumors. Limited data from recent clinical studies demonstrate prolonged progression-free survival of patients who have received intrapleural or intraperitoneal TIL therapy. Investigators are further exploring this in the Fast TIL clinical trial, in which patients’ pleural fluid is being used to create a rapidly expanded cellular product. These cellular therapeutics show strong potential for combating the immunosuppressive tumor environment.

## 7. Conclusions and Future Perspectives

Peritoneal carcinomatosis (PC) and malignant pleural effusion (MPE) occur when cancers metastasize to or originate in the pleural and peritoneal cavities, which constitute a unique environment adapted to the maintenance and repair of the mesothelial lining, created by resident T-cells, macrophages, and mesothelial cells. The incursion of neoplastic cells into this milieu amplifies the inflammatory and immunosuppressive characteristics of this environment. The result is often a serous effusion with an influx of immune cells that further exacerbate the problem through the secretion of immunosuppressive cytokines. These include IL-10, TGFβ, and IL-1RA, and amplification of the IL-6/soluble IL-6Rα axis. This maladaptive cavitary environment suppresses anti-tumor immunity and promotes tumor EMT with attendant increases in motility and drug resistance. This environment is largely responsible for the poor prognosis associated with cavitary malignancies. If there is a positive aspect to this biological paradigm, it is that T effector memory and central memory cells are present among the cavitary-infiltrating immune cells. Their potent anti-tumor activity is demonstrable when removed from the cavitary environment and stimulated Ex Vivo.

Interest in cancer immunotherapy for treatment-resistant cancers has been renewed by several important discoveries and technologies, including targetable ICM and the ability to identify immunogenic tumor neoantigens in individual tumors. The goals of cancer immunotherapy remain prospectively identifying and expanding the pool of patients who will respond to treatment, and streamlining manufacturing of cost-effective personalized therapeutics. Emerging treatments for PC and MPE include cellular therapeutics like CAR-T and TIL, immune checkpoint inhibition, bispecific antibodies, and oncolytic viruses. Many of these treatments can take advantage of intracavitary administration to concentrate the therapeutic in the immediate tumor environment and reduce systemic toxicity. Preclinical and clinical data justify continued development of biomarkers identifying responsive patients, drugs targeting the PC and MPE environment, and exploiting the immunologically rich cellular infiltrate.

## Figures and Tables

**Table 1:** Current ongoing immune-oncology trials for the treatment of MPE, MA, or PC diagnoses. Only actively enrolling clinical trials have been included. Source: clinicaltrials.gov (accessed 23 September 2025).

Identifier	Cavitary Environment	Investigational Treatment	Target	Trial Phase	Study Location
NCT06740019	MPE	JMKX000197	STING (Endoplasmic Reticulum IFN stimulator)	I	Beijing, China
NCT05268172	MPE, MA	T Cells in combination with IFNγ	ICAM-1, PD-L1, PD-1	I	Wuxi, Jiangsu, China
NCT06016179	MPE, MA	Tocilizumab	IL-6Rα	I	Pittsburgh, PA, United States
NCT05477927	MPE, MA	Dual-targeting VEGFR1/PD-L1 CAR-T	VEGFR1, PD-L1	I	Chengdu, Sichuan, China
NCT06726564	MPE	MT027	B7H3	I	Beijing, China
NCT07090525	MPE	Bevacizumab	VEGF	II	Qingdao, Shandong, China
NCT04684459	MPE, MA	Dual-targeting HER-2/PD-L1 CAR-T	HER2, PD-L1	I	Chengdu, Sichuan, China
NCT04919629	MPE, MA	APL-2, Pembrolizumab, Bevacizumab	C3 Protein	II	Buffalo, NY, United States
NCT06769295	MPE	AK112	PD-1, VEGF	II	Sichuan, Chengdu, China
NCT05700656	PC	Galunisertib	TGFβ	I, II	Amsterdam, The Netherlands
NCT05801783	PC	R130	CD3 scFv/CD86/PD1/HSV2-US11	I	Shanghai, China
NCT03252938	PC	IMP321	PD-L1	I	Germany
NCT06623396	PC	MSLN-targeted CAR-T	Mesothelin	I	New Jersey, United States
NCT06433869	MA	Bevacizumab, Serplulimab, rmhTNF-NC	VEGF, TNF, PD-1	II	Guangzhou, China
NCT06200376	MA	T3011	PD-1, IL-12	I	Chengdu, Sichuan, China
NCT06759064	MA	Sintilimab	PD-1	I, II	Jinan, Shandong, China
NCT06046963	PC, MA	Sintilimab	PD-1	II	Hangzhou, Zhejiang, China
NCT05438459	MA	GAIA-102	NK Cells	I, II	Fukuoka, Japan

## Data Availability

No new data were created or analyzed in this study. Data sharing is not applicable to this article.

## Abbreviations

ACT	Adoptive cellular therapy
B-ALL	B-cell acute lymphoblastic leukemia
Breg	Regulatory B cells
CAF	Cancer-associated fibroblast
CAR	chimeric antigen receptors
CAR-T	Chimeric antigen receptor T cell
CEA	Carcinoembronic antigen
CLL	Chronic lymphocytic leukemia
CRS	Cytokine release syndrome
ECM	Extracellular matrix
EGF	Epidermal growth factor
EMT	Epithelial-to-mesenchymal transition
FDA	Food and Drug Administration
FOXP1	Forkhead box protein 1
HER2	Human epidermal growth factor receptor 2
HIPEC	Hyperthermic intraperitoneal chemotherapy
ICM	Immune checkpoint molecules
IFN	Interferon
IL	Interleukin
IND	Investigational new drug
IPC	Indwelling pleural catheter
LDC	Lymphodepleting chemotherapy
MA	Malignant ascites
mAb	Monoclonal antibody
MDSC	Myeloid-derived suppressor cells
MHC	Major histocompatibility complex
MMT	Mesothelial-to-mesenchymal transition
MPE	Malignant pleural effusion
MPM	Malignant pleural mesothelioma
NK	Natural killer
NSCLC	Non-small cell lung cancer
PAMP	Pathogen-associated molecular patterns
PC	Peritoneal carcinomatosis
TCR	T cell receptor
TGF	Transforming growth factor
TIL	Tumor-infiltrating lymphocytes
TNF	Tumor necrosis factor
Treg	Regulatory T cells
VEGF	Vascular endothelial growth factor

## References

[1] Guan X. (2015). Cancer metastases: Challenges and opportunities. Acta Pharm. Sin. B.

[2] Chu D.Z., Lang N.P., Thompson C., Osteen P.K., Westbrook K.C. (1989). Peritoneal carcinomatosis in nongynecologic malignancy. A prospective study of prognostic factors. Cancer.

[3] Kerscher A.G., Chua T.C., Gasser M., Maeder U., Kunzmann V., Isbert C., Germer C.T., Pelz J.O.W. (2013). Impact of peritoneal carcinomatosis in the disease history of colorectal cancer management: A longitudinal experience of 2406 patients over two decades. Br. J. Cancer.

[4] Bashour S.I., Mankidy B.J., Lazarus D.R. (2022). Update on the diagnosis and management of malignant pleural effusions. Respir. Med..

[5] Donnenberg V.S., Luketich J.D., Sultan I., Lister J., Bartlett D.L., Ghosh S., Donnenberg A.D. (2023). A maladaptive pleural environment suppresses preexisting anti-tumor activity of pleural infiltrating T cells. Front. Immunol..

[6] Lewis C.R., Dadgar N., Yellin S.A., Donnenberg V.S., Donnenberg A.D., Bartlett D.L., Allen C.J., Wagner P.L. (2023). Regional Immunotherapy for Peritoneal Carcinomatosis in Gastroesophageal Cancer: Emerging Strategies to Re-Condition a Maladaptive Tumor Environment. Cancers.

[7] Ornella M.S.C., Badrinath N., Kim K.-A., Kim J.H., Cho E., Hwang T.-H., Kim J.-J. (2023). Immunotherapy for Peritoneal Carcinomatosis: Challenges and Prospective Outcomes. Cancers.

[8] Terri M., Trionfetti F., Montaldo C., Cordani M., Tripodi M., Lopez-Cabrera M., Strippoli R. (2021). Mechanisms of Peritoneal Fibrosis: Focus on Immune Cells–Peritoneal Stroma Interactions. Front. Immunol..

[9] Rudralingam V., Footitt C., Layton B. (2017). Ascites matters. Ultrasound.

[10] Szadkowska M.A., Pałucki J., Cieszanowski A. (2023). Diagnosis and treatment of peritoneal carcinomatosis—A comprehensive overview. Pol. J. Radiol..

[11] Dadgar N., Sherry C., Zimmerman J., Park H., Lewis C., Donnenberg A., Zaidi A.H., Fan Y., Xiao K., Bartlett D. (2024). Targeting interleukin-6 as a treatment approach for peritoneal carcinomatosis. J. Transl. Med..

[12] Sangisetty S.L., Miner T.J. (2012). Malignant ascites: A review of prognostic factors, pathophysiology and therapeutic measures. World J. Gastrointest. Surg..

[13] Berger J.M., Preusser M., Berghoff A.S., Bergen E.S. (2023). Malignant ascites: Current therapy options and treatment prospects. Cancer Treat. Rev..

[14] Donnenberg A.D., Luketich J.D., Donnenberg V.S. (2019). Secretome of pleural effusions associated with non-small cell lung cancer (NSCLC) and malignant mesothelioma: Therapeutic implications. Oncotarget.

[15] Gayen S. (2022). Malignant Pleural Effusion: Presentation, Diagnosis, and Management. Am. J. Med..

[16] Porcel J.M., Light R.W. (2006). Diagnostic Approach to Pleural Effusion in Adults. Am. Fam. Physician.

[17] Musso V., Diotti C., Palleschi A., Tosi D., Aiolfi A., Mendogni P. (2021). Management of Pleural Effusion Secondary to Malignant Mesothelioma. J. Clin. Med..

[18] Gonnelli F., Hassan W., Bonifazi M., Pinelli V., Bedawi E.O., Porcel J.M., Rahman N.M., Mei F. (2024). Malignant pleural effusion: Current understanding and therapeutic approach. Respir. Res..

[19] Baguneid A., Wijayaratne T., Aujayeb A., Panchal R. (2025). The Evolution of the Indwelling Pleural Catheter. Pulm. Ther..

[20] Zamboni M.M., da Silva C.T., Baretta R., Cunha E.T., Cardoso G.P. (2015). Important prognostic factors for survival in patients with malignant pleural effusion. BMC Pulm. Med..

[21] Rathod S., Aranda F., Berraondo P., Galluzzi L. (2022). Chapter Two—T cells in the peritoneum. International Review of Cell and Molecular Biology.

[22] Budna J., Kaczmarek M., Kolecka-Bednarczyk A., Spychalski Ł., Zawierucha P., Goździk-Spychalska J., Nowicki M., Batura-Gabryel H., Sikora J. (2018). Enhanced Suppressive Activity of Regulatory T Cells in the Microenvironment of Malignant Pleural Effusions. J. Immunol. Res..

[23] Kubicka U., Olszewski W.L., Tarnowski W., Bielecki K., Ziółkowska A., Wierzbicki Z. (1996). Normal human immune peritoneal cells: Subpopulations and functional characteristics. Scand. J. Immunol..

[24] Noppen M., De Waele M., Li R., Gucht K.V., D’Haese J., Gerlo E., Vincken W. (2000). Volume and cellular content of normal pleural fluid in humans examined by pleural lavage. Am. J. Respir. Crit. Care Med..

[25] Liu T., Liu F., Peng L.W., Chang L., Jiang Y.M. (2018). The Peritoneal Macrophages in Inflammatory Diseases and Abdominal Cancers. Oncol. Res..

[26] Kaczmarek M., Sikora J. (2012). Macrophages in malignant pleural effusions—Alternatively activated tumor associated macrophages. Contemp. Oncol..

[27] Yamaguchi T., Fushida S., Yamamoto Y., Tsukada T., Kinoshita J., Oyama K., Miyashita T., Tajima H., Ninomiya I., Munesue S. (2016). Tumor-associated macrophages of the M2 phenotype contribute to progression in gastric cancer with peritoneal dissemination. Gastric Cancer.

[28] Principe N., Kidman J., Lake R.A., Lesterhuis W.J., Nowak A.K., McDonnell A.M., Chee J. (2021). Malignant Pleural Effusions—A Window Into Local Anti-Tumor T Cell Immunity?. Front. Oncol..

[29] Wang F., Yang L., Gao Q., Huang L., Wang L., Wang J., Wang S., Zhang B., Zhang Y. (2015). CD163+CD14+ macrophages, a potential immune biomarker for malignant pleural effusion. Cancer Immunol. Immunother..

[30] Liu C.-Y., Xu J.-Y., Shi X.-Y., Huang W., Ruan T.-Y., Xie P., Ding J.-L. (2013). M2-polarized tumor-associated macrophages promoted epithelial–mesenchymal transition in pancreatic cancer cells, partially through TLR4/IL-10 signaling pathway. Lab. Investig..

[31] Jie X.-X., Zhang X.-Y., Xu C.-J. (2017). Epithelial-to-mesenchymal transition, circulating tumor cells and cancer metastasis: Mechanisms and clinical applications. Oncotarget.

[32] Song K.-A., Faber A.C. (2019). Epithelial-to-mesenchymal transition and drug resistance: Transitioning away from death. J. Thorac. Dis..

[33] Szabo P.M., Vajdi A., Kumar N., Tolstorukov M.Y., Chen B.J., Edwards R., Ligon K.L., Chasalow S.D., Chow K.-H., Shetty A. (2023). Cancer-associated fibroblasts are the main contributors to epithelial-to-mesenchymal signatures in the tumor microenvironment. Sci. Rep..

[34] Mulet M., Osuna-Gomez R., Zamora C., Porcel J.M., Nieto J.C., Perea L., Pajares V., Muñoz-Fernandez A.M., Calvo N., Sorolla M.A. (2022). Influence of Malignant Pleural Fluid from Lung Adenocarcinoma Patients on Neutrophil Response. Cancers.

[35] Gabrilovich D.I. (2017). Myeloid-derived suppressor cells. Cancer Immunol. Res..

[36] Nair R., Somasundaram V., Kuriakose A., Krishn S.R., Raben D., Salazar R., Nair P. (2025). Deciphering T-cell exhaustion in the tumor microenvironment: Paving the way for innovative solid tumor therapies. Front. Immunol..

[37] Sen D.R., Kaminski J., Barnitz R.A., Kurachi M., Gerdemann U., Yates K.B., Tsao H.W., Godec J., LaFleur M.W., Brown F.D. (2016). The epigenetic landscape of T cell exhaustion. Science.

[38] Wherry E.J., Ha S.-J., Kaech S.M., Haining W.N., Sarkar S., Kalia V., Subramaniam S., Blattman J.N., Barber D.L., Ahmed R. (2007). Molecular Signature of CD8^+^ T Cell Exhaustion during Chronic Viral Infection. Immunity.

[39] van der Heide V., Humblin E., Vaidya A., Kamphorst A.O. (2022). Advancing beyond the twists and turns of T cell exhaustion in cancer. Sci. Transl. Med..

[40] Doering Travis A., Crawford A., Angelosanto Jill M., Paley Michael A., Ziegler Carly G., Wherry E.J. (2012). Network Analysis Reveals Centrally Connected Genes and Pathways Involved in CD8^+^ T Cell Exhaustion versus Memory. Immunity.

[41] Pearce E.L. (2010). Metabolism in T cell activation and differentiation. Curr. Opin. Immunol..

[42] Chapman N.M., Boothby M.R., Chi H. (2020). Metabolic coordination of T cell quiescence and activation. Nat. Rev. Immunol..

[43] Sprent J., Surh C.D. (2011). Normal T cell homeostasis: The conversion of naive cells into memory-phenotype cells. Nat. Immunol..

[44] Hamilton S.E., Jameson S.C. (2012). CD8 T cell quiescence revisited. Trends Immunol..

[45] Allam A., Conze D.B., Giardino Torchia M.L., Munitic I., Yagita H., Sowell R.T., Marzo A.L., Ashwell J.D. (2009). The CD8^+^ memory T-cell state of readiness is actively maintained and reversible. Blood.

[46] De Silva P., Garaud S., Solinas C., de Wind A., Van den Eyden G., Jose V., Gu-Trantien C., Migliori E., Boisson A., Naveaux C. (2019). FOXP1 negatively regulates tumor infiltrating lymphocyte migration in human breast cancer. EBioMedicine.

[47] Tu E., Chia C.P.Z., Chen W., Zhang D., Park S.A., Jin W., Wang D., Alegre M.-L., Zhang Y.E., Sun L. (2018). T Cell Receptor-Regulated TGF-beta Type I Receptor Expression Determines T Cell Quiescence and Activation. Immunity.

[48] Donnenberg V.S., Luketich J.D., Popov B., Bartlett D.L., Donnenberg A.D. (2024). A common secretomic signature across epithelial cancers metastatic to the pleura supports IL-6 axis therapeutic targeting. Front. Immunol..

[49] Wagner P.L., Knotts C.M., Donnenberg V.S., Dadgar N., Cruz Pico C.X., Xiao K., Zaidi A., Schifman S.C., Allen C.J., Donnenberg A.D. (2024). Characterizing the Immune Environment in Peritoneal Carcinomatosis: Insights for Novel Immunotherapy Strategies. Ann. Surg. Oncol..

[50] Hu C.Y., Zhang Y.H., Wang T., Chen L., Gong Z.H., Wan Y.S., Li Q.J., Li Y.S., Zhu B. (2016). Interleukin-2 reverses CD8^+^ T cell exhaustion in clinical malignant pleural effusion of lung cancer. Clin. Exp. Immunol..

[51] Chen J., Wei Y., Yang W., Huang Q., Chen Y., Zeng K., Chen J. (2022). IL-6: The Link Between Inflammation, Immunity and Breast Cancer. Front. Oncol..

[52] Donnenberg A.D., Luketich J.D., Dhupar R., Donnenberg V.S. (2019). Treatment of malignant pleural effusions: The case for localized immunotherapy. J. Immunother. Cancer.

[53] Heikkilä K., Ebrahim S., Lawlor D.A. (2008). Systematic review of the association between circulating interleukin-6 (IL-6) and cancer. Eur. J. Cancer.

[54] Lippitz B.E. (2013). Cytokine patterns in patients with cancer: A systematic review. Lancet Oncol..

[55] Shkhyan R., Flynn C., Lamoure E., Sarkar A., Van Handel B., Li J., York J., Banks N., Van der Horst R., Liu N.Q. (2023). Inhibition of a signaling modality within the gp130 receptor enhances tissue regeneration and mitigates osteoarthritis. Sci. Transl. Med..

[56] Steensberg A., Fischer C.P., Keller C., Møller K., Pedersen B.K. (2003). IL-6 enhances plasma IL-1ra, IL-10, and cortisol in humans. Am. J. Physiol.-Endocrinol. Metabolism..

[57] Wertel I., Suszczyk D., Pawłowska A., Bilska M., Chudzik A., Skiba W., Paduch R., Kotarski J. (2020). Prognostic and Clinical Value of Interleukin 6 and CD45^+^CD14^+^ Inflammatory Cells with PD-L1^+^/PD-L2^+^ Expression in Patients with Different Manifestation of Ovarian Cancer. J. Immunol. Res..

[58] Sato T., Terai M., Tamura Y., Alexeev V., Mastrangelo M.J., Selvan S.R. (2011). Interleukin 10 in the tumor microenvironment: A target for anticancer immunotherapy. Immunol. Res..

[59] Mannino M.H., Zhu Z., Xiao H., Bai Q., Wakefield M.R., Fang Y. (2015). The paradoxical role of IL-10 in immunity and cancer. Cancer Lett..

[60] Dennis K.L., Blatner N.R., Gounari F., Khazaie K. (2013). Current status of IL-10 and regulatory T-cells in cancer. Curr. Opin. Oncol..

[61] Iyer S.S., Cheng G. (2012). Role of Interleukin 10 Transcriptional Regulation in Inflammation and Autoimmune Disease. Crit. Rev.™ Immunol..

[62] Nixon B.G., Gao S., Wang X., Li M.O. (2023). TGFβ control of immune responses in cancer: A holistic immuno-oncology perspective. Nat. Rev. Immunol..

[63] Batlle E., Massagué J. (2019). Transforming Grown Factor-β Signaling in Immunity and Cancer. Immunity.

[64] Xu J., Lamouille S., Derynck R. (2009). TGF-β-induced epithelial to mesenchymal transition. Cell Res..

[65] Stockhammer P., Ploenes T., Theegarten D., Schuler M., Maier S., Aigner C., Hegedus B. (2020). Detection of TGF-β in pleural effusions for diagnosis and prognostic stratification of malignant pleural mesothelioma. Lung Cancer.

[66] Jain A., Song R., Wakeland E.K., Pasare C. (2018). T cell-intrinsic IL-1R signaling licenses effector cytokine production by memory CD4 T cells. Nat. Commun..

[67] Togashi Y., Shitara K., Nishikawa H. (2019). Regulatory T cells in cancer immunosuppression—Implications for anticancer therapy. Nat. Rev. Clin. Oncol..

[68] Budna J., Spychalski Ł., Kaczmarek M., Frydrychowicz M., Goździk-Spychalska J., Batura-Gabryel H., Sikora J. (2017). Regulatory T cells in malignant pleural effusions subsequent to lung carcinoma and their impact on the course of the disease. Immunobiology.

[69] Landskron J., Helland Ø., Torgersen K.M., Aandahl E.M., Gjertsen B.T., Bjørge L., Taskén K. (2014). Activated regulatory and memory T-cells accumulate in malignant ascites from ovarian carcinoma patients. Cancer Immunol. Immunother. CII.

[70] Nowatzky J., Stagnar C., Manches O. (2019). OMIP-053: Identification, Classification, and Isolation of Major FoxP3 Expressing Human CD4(+) Treg Subsets. Cytom. A.

[71] Glass M.C., Glass D.R., Oliveria J.P., Mbiribindi B., Esquivel C.O., Krams S.M., Bendall S.C., Martinez O.M. (2022). Human IL-10-producing B cells have diverse states that are induced from multiple B cell subsets. Cell Rep..

[72] Takahashi K., Kurashina K., Yamaguchi H., Kanamaru R., Ohzawa H., Miyato H., Saito S., Hosoya Y., Lefor A.K., Sata N. (2022). Altered intraperitoneal immune microenvironment in patients with peritoneal metastases from gastric cancer. Front. Immunol..

[73] Popowicz N., Cheah H.M., Gregory C., Miranda A., Dick I.M., Lee Y.C.G., Creaney J. (2021). Neutrophil-to-lymphocyte ratio in malignant pleural fluid: Prognostic significance. PLoS ONE.

[74] Ge S., Zhao Y., Liang J., He Z., Li K., Zhang G., Hua B., Zheng H., Guo Q., Qi R. (2024). Immune modulation in malignant pleural effusion: From microenvironment to therapeutic implications. Cancer Cell Int..

[75] Gabrilovich D.I., Ostrand-Rosenberg S., Bronte V. (2012). Coordinated regulation of myeloid cells by tumours. Nat. Rev. Immunol..

[76] Tobin R.P., Jordan K.R., Kapoor P., Spongberg E., Davis D., Vorwald V.M., Couts K.L., Gao D., Smith D.E., Borgers J.S.W. (2019). IL-6 and IL-8 Are Linked With Myeloid-Derived Suppressor Cell Accumulation and Correlate With Poor Clinical Outcomes in Melanoma Patients. Front. Oncol..

[77] Sugita Y., Yamashita K., Fujita M., Saito M., Yamada K., Agawa K., Watanabe A., Fukuoka E., Hasegawa H., Kanaji S. (2021). CD244^+^ polymorphonuclear myeloid-derived suppressor cells reflect the status of peritoneal dissemination in a colon cancer mouse model. Oncol. Rep..

[78] Werb Z., Lu P. (2015). The Role of Stroma in Tumor Development. Cancer J..

[79] Connolly J.L., Schnitt S.J., Wang H.H., Longtine J.A., Dvorak A., Dvorak H.F. (2003). Tumor Structure and Tumor Stroma Generation. Holland-Frei Cancer Medicine.

[80] Karpathiou G., Péoc’h M., Sundaralingam A., Rahman N., Froudarakis M.E. (2022). Inflammation of the Pleural Cavity: A Review on Pathogenesis, Diagnosis and Implications in Tumor Pathophysiology. Cancers.

[81] Markov A.G., Voronkova M.A., Volgin G.N., Yablonsky P.K., Fromm M., Amasheh S. (2011). Tight junction proteins contribute to barrier properties in human pleura. Respir. Physiol. Neurobiol..

[82] Rakina M., Kazakova A., Villert A., Kolomiets L., Larionova I. (2022). Spheroid Formation and Peritoneal Metastasis in Ovarian Cancer: The Role of Stromal and Immune Components. Int. J. Mol. Sci..

[83] Rynne-Vidal A., Jiménez-Heffernan J.A., Fernández-Chacón C., López-Cabrera M., Sandoval P. (2015). The Mesothelial Origin of Carcinoma Associated-Fibroblasts in Peritoneal Metastasis. Cancers.

[84] Lu H., Clauser K.R., Tam W.L., Frose J., Ye X., Eaton E.N., Reinhardt F., Donnenberg V.S., Bhargava R., Carr S.A. (2014). A breast cancer stem cell niche supported by juxtacrine signalling from monocytes and macrophages. Nat. Cell Biol..

[85] Yin T., Wang G., He S., Shen G., Su C., Zhang Y., Wei X., Ye T., Li L., Yang S. (2016). Malignant Pleural Effusion and ascites Induce Epithelial-Mesenchymal Transition and Cancer Stem-like Cell Properties via the Vascular Endothelial Growth Factor (VEGF)/Phosphatidylinositol 3-Kinase (PI3K)/Akt/Mechanistic Target of Rapamycin (mTOR) Pathway. J. Biol. Chem..

[86] Miao Z.-F., Zhao T.-T., Wang Z.-N., Miao F., Xu Y.-Y., Mao X.-Y., Gao J., Wu J.-H., Liu X.-Y., You Y. (2014). Transforming growth factor-beta1 signaling blockade attenuates gastric cancer cell-induced peritoneal mesothelial cell fibrosis and alleviates peritoneal dissemination both in vitro and in vivo. Tumour Biol..

[87] Ito M., Nakano M., Ariyama H., Yamaguchi K., Tanaka R., Semba Y., Sugio T., Miyawaki K., Kikushige Y., Mizuno S. (2022). Macrophages are primed to transdifferentiate into fibroblasts in malignant ascites and pleural effusions. Cancer Lett..

[88] Lorenc E., Varinelli L., Chighizola M., Brich S., Pisati F., Guaglio M., Baratti D., Deraco M., Gariboldi M., Podestà A. (2023). Correlation between biological and mechanical properties of extracellular matrix from colorectal peritoneal metastases in human tissues. Sci. Rep..

[89] Berek J.S., Hacker N.F., Lichtenstein A., Jung T., Spina C., Knox R.M., Brady J., Greene T., Ettinger L.M., Lagasse L.D. (1985). Intraperitoneal recombinant alpha-interferon for “salvage” immunotherapy in stage III epithelial ovarian cancer: A Gynecologic Oncology Group Study. Cancer Res..

[90] Toge T., Yamada H., Aratani K., Kameda A., Kuroi K., Hisamatsu K., Hattori T. (1985). Effects of intraperitoneal administration of OK-432 for patients with advanced cancer. Jpn. J. Surg..

[91] Knisely A., Hinchcliff E., Fellman B., Mosley A., Lito K., Hull S., Westin S.N., Sood A.K., Schmeler K.M., Taylor J.S. (2024). Phase 1b study of intraperitoneal ipilimumab and nivolumab in patients with recurrent gynecologic malignancies with peritoneal carcinomatosis. Med.

[92] Hamanishi J., Mandai M., Ikeda T., Minami M., Kawaguchi A., Murayama T., Kanai M., Mori Y., Matsumoto S., Chikuma S. (2015). Safety and Antitumor Activity of Anti-PD-1 Antibody, Nivolumab, in Patients With Platinum-Resistant Ovarian Cancer. J. Clin. Oncol..

[93] Kang Y.-K., Boku N., Satoh T., Ryu M.-H., Chao Y., Kato K., Chung H.C., Chen J.-S., Muro K., Kang W.K. (2017). Nivolumab in patients with advanced gastric or gastro-oesophageal junction cancer refractory to, or intolerant of, at least two previous chemotherapy regimens (ONO-4538-12, ATTRACTION-2): A randomised, double-blind, placebo-controlled, phase 3 trial. Lancet.

[94] Zhang L., Mai W., Jiang W., Geng Q. (2020). Sintilimab: A Promising Anti-Tumor PD-1 Antibody. Front. Oncol..

[95] Lv T., Wu G., Song X., Li X., Zhang J., Song Y. (2021). P16.05 Exploratory Study of Sintilimab Intrapleural Therapy for NSCLC-Mediated Malignant Pleural Effusion. J. Thorac. Oncol..

[96] Tsimafeyeu I., Goutnik V., Shrainer I., Kosyrev V., Bondarenko A., Utyashev I. (2023). Multicenter phase 2 study of intrapleural nivolumab in patients with metastatic non-small cell lung cancer and pleural effusion. Am. J. Cancer Res..

[97] Tsimafeyeu I., Goutnik V., Shrainer I., Kosyrev V., Bondarenko A., Utyashev I. (2024). Intrapleural nivolumab in cancer patients with pleural effusion. J. Cancer Res. Ther..

[98] Hossen M.M., Ma Y., Yin Z., Xia Y., Du J., Huang J.Y., Huang J.J., Zou L., Ye Z., Huang Z. (2023). Current understanding of CTLA-4: From mechanism to autoimmune diseases. Front. Immunol..

[99] Zhao Y., Yang W., Huang Y., Cui R., Li X., Li B. (2018). Evolving Roles for Targeting CTLA-4 in Cancer Immunotherapy. Cell. Physiol. Biochem..

[100] Kooshkaki O., Derakhshani A., Hosseinkhani N., Torabi M., Safaei S., Brunetti O., Racanelli V., Silvestris N., Baradaran B. (2020). Combination of Ipilimumab and Nivolumab in Cancers: From Clinical Practice to Ongoing Clinical Trials. Int. J. Mol. Sci..

[101] Murthy P., Ekeke C.N., Russell K.L., Butler S.C., Wang Y., Luketich J.D., Soloff A.C., Dhupar R., Lotze M.T. (2019). Making cold malignant pleural effusions hot: Driving novel immunotherapies. Oncoimmunology.

[102] Finley S.D., Popel A.S. (2013). Effect of Tumor Microenvironment on Tumor VEGF During Anti-VEGF Treatment: Systems Biology Predictions. JNCI J. Natl. Cancer Inst..

[103] Zebrowski B.K., Liu W., Ramirez K., Akagi Y., Mills G.B., Ellis L.M. (1999). Markedly Elevated Levels of Vascular Endothelial Growth Factor in Malignant Ascites. Ann. Surg. Oncol..

[104] Zeng H., Zhang Y., Tan S., Huang Q., Pu X., Tian P., Li Y. (2024). Efficacy of bevacizumab through an indwelling pleural catheter in non-small cell lung cancer patients with symptomatic malignant pleural effusion. BMC Pulm. Med..

[105] Razenberg L.G.E.M., van Gestel Y.R.B.M., Lemmens V.E.P.P., de Hingh I.H.J.T., Creemers G.-J. (2016). Bevacizumab in Addition to Palliative Chemotherapy for Patients With Peritoneal Carcinomatosis of Colorectal Origin: A Nationwide Population-Based Study. Clin. Color. Cancer.

[106] Sjoquist K.M., Espinoza D., Mileshkin L., Ananda S., Shannon C., Yip S., Goh J., Bowtell D., Harrison M., Friedlander M.L. (2021). REZOLVE (ANZGOG-1101): A phase 2 trial of intraperitoneal bevacizumab to treat symptomatic ascites in patients with chemotherapy-resistant, epithelial ovarian cancer. Gynecol. Oncol..

[107] Jordan K., Luetkens T., Gog C., Killing B., Arnold D., Hinke A., Stahl M., Freier W., Rüssel J., Atanackovic D. (2016). Intraperitoneal bevacizumab for control of malignant ascites due to advanced-stage gastrointestinal cancers: A multicentre double-blind, placebo-controlled phase II study—AIO SUP-0108. Eur. J. Cancer.

[108] Ghasemi K., Ghasemi K. (2022). Evaluation of the Tocilizumab therapy in human cancers: Latest evidence and clinical potential. J. Clin. Pharm. Ther..

[109] Park H., Lewis C., Dadgar N., Sherry C., Evans S., Ziobert S., Omstead A., Zaidi A., Xiao K., Ghosh S. (2024). Intra-pleural and intra-peritoneal tocilizumab therapy for managing malignant pleural effusions and ascites: The Regional Immuno-Oncology Trial (RIOT)—2 Study protocol. Surg. Oncol. Insight.

[110] Sun Y., Yu X., Wang X., Yuan K., Wang G., Hu L., Zhang G., Pei W., Wang L., Sun C. (2023). Bispecific antibodies in cancer therapy: Target selection and regulatory requirements. Acta Pharm. Sin. B.

[111] Knödler M., Körfer J., Kunzmann V., Trojan J., Daum S., Schenk M., Kullmann F., Schroll S., Behringer D., Stahl M. (2018). Randomised phase II trial to investigate catumaxomab (anti-EpCAM × anti-CD3) for treatment of peritoneal carcinomatosis in patients with gastric cancer. Br. J. Cancer.

[112] Burges A., Wimberger P., Kümper C., Gorbounova V., Sommer H., Schmalfeldt B., Pfisterer J., Lichinitser M., Makhson A., Moiseyenko V. (2007). Effective Relief of Malignant Ascites in Patients with Advanced Ovarian Cancer by a Trifunctional Anti-EpCAM × Anti-CD_3_ Antibody: A Phase I/II Study. Clin. Cancer Res..

[113] Heiss M.M., Murawa P., Koralewski P., Kutarska E., Kolesnik O.O., Ivanchenko V.V., Dudnichenko A.S., Aleknaviciene B., Razbadauskas A., Gore M. (2010). The trifunctional antibody catumaxomab for the treatment of malignant ascites due to epithelial cancer: Results of a prospective randomized phase II/III trial. Int. J. Cancer.

[114] Sebastian M., Jaeger M., Kiewe P., Schuette W., Wiewrodt R., Lindhofer H., Mueller B., Friccius-Quecke H., Quecke H.F., Schmittel A. (2007). Effects of the trifunctional antibody catumaxomab (anti-EpCAM × anti-CD_3_) on proliferation and cytokine secretion of immune cells in malignant pleural effusion. J. Clin. Oncol..

[115] Liu R., Xu J., Lin R., Li N., Li G., Zhang T., Zhao J., Li J., Sun M., Wang K. (2024). Updated results of a phase II trial evaluating an anti-EpCAM × anti-CD_3_ bispecific antibody, M701, for the treatment of malignant ascites. Ann. Oncol..

[116] Cai J., Zhang F., Song Z., Jin J., Lv D., Pang W., Yi T., Wang G., Yao J., Wang B. (2024). 1371P An anti-EpCAM × CD_3_ bispecific antibody, M701, for the treatment of malignant pleural effusion in NSCLC patients: Intermediate results of a prospective multicenter phase Ib trial. Ann. Oncol..

[117] Santos Apolonio J., Lima de Souza Gonçalves V., Cordeiro Santos M.L., Silva Luz M., Silva Souza J.V., Rocha Pinheiro S.L., de Souza W.R., Sande Loureiro M., de Melo F.F. (2021). Oncolytic virus therapy in cancer: A current review. World J. Virol..

[118] Guo Z.S., Bartlett D.L. (2014). Oncolytic viruses as platform for multimodal cancer therapeutics: A promising land. Cancer Gene Ther..

[119] Lin D., Shen Y., Liang T. (2023). Oncolytic virotherapy: Basic principles, recent advances and future directions. Signal Transduct. Target. Ther..

[120] McCart J.A., Ward J.M., Lee J., Hu Y., Alexander H.R., Libutti S.K., Moss B., Bartlett D.L. (2001). Systemic cancer therapy with a tumor-selective vaccinia virus mutant lacking thymidine kinase and vaccinia growth factor genes. Cancer Res..

[121] Ge Y., Wang H., Ren J., Liu W., Chen L., Chen H., Ye J., Dai E., Ma C., Ju S. (2020). Oncolytic vaccinia virus delivering tethered IL-12 enhances antitumor effects with improved safety. J. Immunother. Cancer.

[122] Downs-Canner S., Guo Z.S., Ravindranathan R., Breitbach C.J., O’Malley M.E., Jones H.L., Moon A., McCart J.A., Shuai Y., Zeh H.J. (2016). Phase 1 Study of Intravenous Oncolytic Poxvirus (vvDD) in Patients With Advanced Solid Cancers. Mol. Ther..

[123] Gong J., Sachdev E., Mita A.C., Mita M.M. (2016). Clinical development of reovirus for cancer therapy: An oncolytic virus with immune-mediated antitumor activity. World J. Methodol..

[124] Ponce S., Cedres S., Ricordel C., Isambert N., Viteri S., Herrera-Juarez M., Martinez-Marti A., Navarro A., Lederlin M., Serres X. (2023). ONCOS-102 plus pemetrexed and platinum chemotherapy in malignant pleural mesothelioma: A randomized phase 2 study investigating clinical outcomes and the tumor microenvironment. J. Immunother. Cancer.

[125] Wong M.K., Milhem M.M., Sacco J.J., Michels J., In G.K., Munoz Couselo E., Schadendorf D., Beasley G.M., Niu J., Chmielowski B. (2025). RP1 Combined With Nivolumab in Advanced Anti-PD-1-Failed Melanoma (IGNYTE). J. Clin. Oncol..

[126] Lauer U.M., Schell M., Beil J., Berchtold S., Koppenhöfer U., Glatzle J., Königsrainer A., Möhle R., Nann D., Fend F. (2018). Phase I Study of Oncolytic Vaccinia Virus GL-ONC1 in Patients with Peritoneal Carcinomatosis. Clin. Cancer Res..

[127] Weibel S., Hofmann E., Basse-Luesebrink T.C., Donat U., Seubert C., Adelfinger M., Gnamlin P., Kober C., Frentzen A., Gentschev I. (2013). Treatment of malignant effusion by oncolytic virotherapy in an experimental subcutaneous xenograft model of lung cancer. J. Transl. Med..

[128] Giehl E., Kosaka H., Liu Z., Feist M., Kammula U.S., Lotze M.T., Ma C., Guo Z.S., Bartlett D.L. (2021). In Vivo Priming of Peritoneal Tumor-Reactive Lymphocytes with a Potent Oncolytic Virus for Adoptive Cell Therapy. Front. Immunol..

[129] Lee Y.S., Lee W.S., Kim C.W., Lee S.J., Yang H., Kong S.J., Ning J., Yang K.M., Kang B., Kim W.R. (2020). Oncolytic vaccinia virus reinvigorates peritoneal immunity and cooperates with immune checkpoint inhibitor to suppress peritoneal carcinomatosis in colon cancer. J. Immunother. Cancer.

[130] Chee J., Watson M.W., Chopra A., Nguyen B., Cook A.M., Creaney J., Lesterhuis W.J., Robinson B.W., Lee Y.C.G., Nowak A.K. (2018). Tumour associated lymphocytes in the pleural effusions of patients with mesothelioma express high levels of inhibitory receptors. BMC Res. Notes.

[131] Yossef R., Tran E., Deniger D.C., Gros A., Pasetto A., Parkhurst M.R., Gartner J.J., Prickett T.D., Cafri G., Robbins P.F. (2018). Enhanced detection of neoantigen-reactive T cells targeting unique and shared oncogenes for personalized cancer immunotherapy. JCI Insight.

[132] Johnson C.B., May B.R., Riesenberg B.P., Suriano S., Mehrotra S., Garrett-Mayer E., Salem M.L., Jeng E.K., Wong H.C., Paulos C.M. (2018). Enhanced Lymphodepletion Is Insufficient to Replace Exogenous IL2 or IL15 Therapy in Augmenting the Efficacy of Adoptively Transferred Effector CD8^+^ T Cells. Cancer Res..

[133] Freedman R.S., Edwards C.L., Kavanagh J.J., Kudelka A.P., Katz R.L., Carrasco C.H., Atkinson E.N., Scott W., Tomasovic B., Templin S. (1994). Intraperitoneal Adoptive Immunotherapy of Ovarian Carcinoma with Tumor-Infiltrating Lymphocytes and Low-Dose Recombinant Interleukin-2: A Pilot Trial. J. Immunother..

[134] Betof Warner A., Hamid O., Komanduri K., Amaria R., Butler M.O., Haanen J., Nikiforow S., Puzanov I., Sarnaik A., Bishop M.R. (2024). Expert consensus guidelines on management and best practices for tumor-infiltrating lymphocyte cell therapy. J. Immunother. Cancer..

[135] Zacharakis N., Huq L.M., Seitter S.J., Kim S.P., Gartner J.J., Sindiri S., Hill V.K., Li Y.F., Paria B.C., Ray S. (2022). Breast Cancers Are Immunogenic: Immunologic Analyses and a Phase II Pilot Clinical Trial Using Mutation-Reactive Autologous Lymphocytes. J. Clin. Oncol..

[136] Paijens S.T., Vledder A., de Bruyn M., Nijman H.W. (2021). Tumor-infiltrating lymphocytes in the immunotherapy era. Cell. Mol. Immunol..

[137] L’Orphelin J.M., Lancien U., Nguyen J.M., Coronilla F.J.S., Saiagh S., Cassecuel J., Boussemart L., Dompmartin A., Dréno B. (2024). NIVO-TIL: Combination anti-PD-1 therapy and adoptive T-cell transfer in untreated metastatic melanoma: An exploratory open-label phase I trial. Acta Oncol..

[138] Marofi F., Motavalli R., Safonov V.A., Thangavelu L., Yumashev A.V., Alexander M., Shomali N., Chartrand M.S., Pathak Y., Jarahian M. (2021). CAR T cells in solid tumors: Challenges and opportunities. Stem Cell Res. Ther..

[139] Park J.H., Geyer M.B., Brentjens R.J. (2016). CD19-targeted CAR T-cell therapeutics for hematologic malignancies: Interpreting clinical outcomes to date. Blood.

[140] Adusumilli P.S., Zauderer M.G., Rivière I., Solomon S.B., Rusch V.W., O’Cearbhaill R.E., Zhu A., Cheema W., Chintala N.K., Halton E. (2021). A Phase I Trial of Regional Mesothelin-Targeted CAR T-cell Therapy in Patients with Malignant Pleural Disease, in Combination with the Anti–PD-1 Agent Pembrolizumab. Cancer Discov..

[141] Bagley S.J., Logun M., Fraietta J.A., Wang X., Desai A.S., Bagley L.J., Nabavizadeh A., Jarocha D., Martins R., Maloney E. (2024). Intrathecal bivalent CAR T cells targeting EGFR and IL13Ralpha2 in recurrent glioblastoma: Phase 1 trial interim results. Nat. Med..

[142] Morello A., Sadelain M., Adusumilli P.S. (2016). Mesothelin-Targeted CARs: Driving T Cells to Solid Tumors. Cancer Discov..

[143] Dobersberger M., Sumesgutner D., Zajc C.U., Salzer B., Laurent E., Emminger D., Sylvander E., Lehner E., Teufl M., Seigner J. (2024). An engineering strategy to target activated EGFR with CAR T cells. Cell Rep. Methods.

[144] Katz S.C., Point G.R., Cunetta M., Thorn M., Guha P., Espat N.J., Boutros C., Hanna N., Junghans R.P. (2016). Regional CAR-T cell infusions for peritoneal carcinomatosis are superior to systemic delivery. Cancer Gene Ther..

[145] Murad J.P., Kozlowska A.K., Lee H.J., Ramamurthy M., Chang W.-C., Yazaki P., Colcher D., Shively J., Cristea M., Forman S.J. (2018). Effective Targeting of TAG72^+^ Peritoneal Ovarian Tumors via Regional Delivery of CAR-Engineered T Cells. Front. Immunol..

[146] Aujayeb A., Astoul P. (2025). A Diagnostic Approach to Malignant Pleural Mesothelioma. Pulm. Ther..

[147] Chintala N.K., Restle D., Quach H., Saini J., Bellis R., Offin M., Beattie J., Adusumilli P.S. (2021). CAR T-cell therapy for pleural mesothelioma: Rationale, preclinical development, and clinical trials. Lung Cancer.

[148] Frey N., Porter D. (2019). Cytokine Release Syndrome with Chimeric Antigen Receptor T Cell Therapy. Biol. Blood Marrow Transplant..

[149] Chen F., Teachey D.T., Pequignot E., Frey N., Porter D., Maude S.L., Grupp S.A., June C.H., Melenhorst J.J., Lacey S.F. (2016). Measuring IL-6 and sIL-6R in serum from patients treated with tocilizumab and/or siltuximab following CAR T cell therapy. J. Immunol. Methods.

[150] Wang L., Ma N., Okamoto S., Amaishi Y., Sato E., Seo N., Mineno J., Takesako K., Kato T., Shiku H. (2016). Efficient tumor regression by adoptively transferred CEA-specific CAR-T cells associated with symptoms of mild cytokine release syndrome. Oncoimmunology.

[151] Yang Y., Vedvyas Y., Alcaina Y., Trumper S.J., Babu D.S., Min I.M., Tremblay J.M., Shoemaker C.B., Jin M.M. (2024). Affinity-tuned mesothelin CAR T cells demonstrate enhanced targeting specificity and reduced off-tumor toxicity. JCI Insight.

[152] Donnenberg V.S., Lister J., Briedenbaugh C.L., Wagner P., Bartlett D., Donnenberg A.D. (2025). Fast TIL: Rapid Manufacture of an Adoptive Cellular Therapeutic from Pleural Infiltrating T cells for Intrapleural Administration. Cytotherapy.

[153] Chu H., Du F., Gong Z., Lian P., Wang Z., Li P., Hu B., Chi C., Chen J. (2017). Better Clinical Efficiency of TILs for Malignant Pleural Effusion and Ascites than Cisplatin Through Intrapleural and Intraperitoneal Infusion. Anticancer. Res..

